# Venous thromboembolism and persistent pulmonary hypertension in cancer patients: a cross-sectional study

**DOI:** 10.1186/s12959-016-0077-1

**Published:** 2016-02-08

**Authors:** Siegfried Wieshammer, Jens Dreyhaupt, Dirk Müller, Felix Momm, Andreas Jakob

**Affiliations:** Department of Cardiology, Pulmonology and Critical Care Medicine, Offenburg Hospital, Weingartenstrasse 70, D-77654 Offenburg, Germany; Institute of Epidemiology and Medical Biometry, University of Ulm, D-89075 Ulm, Germany; Department of Radiation Oncology, Offenburg Hospital, D-77654 Offenburg, Germany; Department of Medical Oncology, Offenburg Hospital, D-77654 Offenburg, Germany

**Keywords:** Venous thromboembolism, Pulmonary hypertension, Cancer, Heart disease

## Abstract

**Background:**

Cancer patients are at increased risk for venous thromboembolism (VTE).

**Objective:**

This monocenter cross-sectional study prospectively assessed the association between a history of ≥1 VTE episode and the presence of pulmonary hypertension (PH) among cancer patients presenting with pulmonary or cardiac symptoms.

**Methods:**

A consecutive series of 583 patients underwent a diagnostic work-up for heart and lung disease. PH was diagnosed if a patient’s peak systolic pressure gradient across the tricuspid valve was ≥35 mmHg, as measured by echocardiography. Using multiple logistic regression analysis, the association between VTE and PH was assessed, following adjustments for age, the presence of severe airway obstruction, atrial fibrillation and left heart diseases.

**Results:**

The prevalence values for PH (*n* = 90) and a history of VTE (*n* = 72) were 15.4 and 12.3 %, respectively. The median time interval between the first VTE episode and referral was 43 months. The odds of PH was higher in the subgroup with VTE (19/72; 26.4 %) than that without VTE (71/511; 13.9 %) in the unadjusted analysis [odds ratio (OR) 2.2, 95 % confidence interval (CI) 1.2, 4.0] and the adjusted model [OR 2.4, 95 % CI 1.2, 4.5]. The risk of PH did not depend on the time interval between VTE and referral. Older age and the presence of severe airway obstruction, atrial fibrillation, and left heart diseases were also associated with an increased odds of PH.

**Conclusion:**

In cancer patients presenting with cardiac or pulmonary symptoms, previous VTE is associated with an increased risk of persistent PH.

## Introduction

Cancer is a risk factor for venous thromboembolism (VTE), which comprises deep vein thrombosis (DVT) and pulmonary embolism (PE) [1–4]. A retrospective cohort study suggested that cancer predisposes patients to developing chronic thromboembolic pulmonary hypertension (CTPH); therefore, the diagnosis of pulmonary hypertension (PH) should prompt a search for CTPH [5]. The presence of PH may often be overlooked among cancer patients because fatigue, exercise intolerance and general clinical deterioration are commonly reported in both conditions. The objective of this study was to determine the impact of previous VTE on the risk of PH in patients referred for evaluation by a pulmonologist or cardiologist because of symptoms suggestive of either lung or heart disease.

## Patients and methods

### Patients

This monocenter cross-sectional study prospectively included 586 consecutive patients with either a history of cancer or active malignant disease who were referred from primary care, oncology or radio-oncology to the pulmonology or cardiology services of an academic teaching hospital from May 2007 to October 2014 for dyspnea, cough, chest pain, pulse irregularities, or exercise intolerance. All but 8 patients were outpatients and none was confined to bed.

Recruitment was stopped after the size of the subgroup with the rarer of the two outcomes (*i.e.*, presence of PH) was well above the minimum sample size as estimated from the rule of 10 outcomes per predictor variable in logistic regression [6]. According to this rule, the sample size of the PH group had to comprise ≥70 subjects to avoid over-fitting because 7 predictor variables were entered into the model.

All patients agreed to participate. We excluded 3 cases because of missing data; thus, 583 patients with 655 malignancies were available for the final analysis. Of 72 patients with multiple tumors, 61 had different tumors. All patients were interviewed for a history of VTE in a standardized manner. The patient’s medical records were reviewed to verify all self-reported VTE and tumor diagnoses that were made at the authors' or other institutions. All patients had symptoms consistent with DVT or PE. The PE group did not include patients with incidental PE discovered during routine imaging studies [7]. A VTE was defined as cancer-related if it occurred in the presence of active malignant disease (see Table 1 for definition) or if a new diagnosis of active malignant disease was made over the following year. The VTE patients were not routinely screened for hereditary thrombophilia.

**Table 1:** Demographic and clinical characteristics of patients with and without venous thromboembolism (VTE)

	PE *n* = 48	VTE *n* = 72	No VTE *n* = 511	*p*-value*
Male:female	(47.9):(52.1)	(45.8):(54.2)	(54.4):(45.6)	0.17^a^
Age^x^, years	71.3 ± 9.6	70.3 ± 9.8	68.1 ± 11.2	0.11^b^
Active malignant disease	20/48 (41.7)	30/72 (41.7)	192/511 (37.6)	0.50^a^
Heart disease	25/48 (52.1)	35/72 (48.6)	197/511 (38.6)	0.10^a^
Impaired left ventricular systolic function	3/48 (6.3)	4/72 (5.6)	31/511 (6.1)	1.00^c^
Atrial fibrillation	5/48 (10.4)	8/72 (11.1)	83/511 (16.2)	0.26^a^
Significant aortic or mitral valve disease	5/48 (10.4)	6/72 (8.3)	40/511 (7.8)	0.88^a^
Left ventricular hypertrophy	7/48 (14.6)	14/72 (19.4)	76/511 (14.9)	0.31^a^
No: mild to moderate: moderately severe: ≥ severe airway obstruction	(63.0):(21.7):(10.9):(4.4)	(70.0):(18.6):(8.6):(2.9)	(62.5):(13.8):(13.0):(10.7)	0.10^a^
Pulmonary hypertension	16/48 (33.3)	19/72 (26.4)	71/511 (13.9)	<0.01^a^

*VTE *versus* No VTE subgroup

Figures in parenthesis are percentages; ^x^mean ± standard deviation; ^a^χ^2^-Test; ^b^t-test; ^c^Fisher’s exact test

Active malignant disease at the time of referral: residual tumor or time interval between surgery/end of chemotherapy/radiotherapy and referral <3 months

Data from a subgroup of the study cohort were published in a previous paper [8]. This study was approved by the ethics committee of the Baden-Württemberg State Chamber of Physicians and informed consent was obtained.

### Diagnostic procedures

The patients underwent an examination for heart and lung disease that included electrocardiography, echocardiography, and pulmonary function testing, as previously described [8]. Further tests were done when clinically indicated. The cardiologists who did the echocardiograms were unaware of the aim of this study.

A diagnosis of PH was made if the peak systolic pressure gradient across the tricuspid valve was ≥35 mmHg, as measured by continuous-wave Doppler echocardiography. Heart disease was diagnosed based on the presence of at least one of the following findings: *(a)* impaired left ventricular systolic function [ejection fraction <50 %, as determined using the biplane modified Simpson’s rule]; *(b)* atrial fibrillation; *(c)* significant valvular heart disease [mild aortic stenosis (mean pressure gradient ≥20 mmHg), moderate aortic regurgitation, moderate mitral regurgitation, or mild mitral stenosis]; and *(d)* left ventricular hypertrophy [end-diastolic septal wall thickness ≥12 mm]. Severe airway obstruction was diagnosed if the forced expiratory volume in 1 s (FEV1)/forced vital capacity (FVC) ratio was <0.70 and if the FEV1 was <50 % of predicted. The degree of obstruction was adjusted for the degree of restriction among the patients with mixed obstructive-restrictive lung disease [9].

### Data analysis

Logistic regression analysis was used to evaluate the association, given as an odds ratio (OR) and a 95 % confidence interval (CI), between a history of VTE (PE) as the primary predictor variable and the presence of PH as the binary outcome. This association was tested in both unadjusted and adjusted models accounting for age, the presence of severe airway obstruction and the above-mentioned cardiac disorders *(a-d)* as prospectively selected disturbance variables. The primary predictor variable and the 6 disturbance variables were treated as equally weighted and independent covariates. In compliance with the above-mentioned 1 in 10 rule, the odds for PH was then additionally adjusted for the presence of active malignant disease at the time of referral [6]. To assess whether the effect of VTE on the odds of PH was time-dependent, the time interval from the first VTE episode to referral was entered into the model in a second step. Given the exploratory nature of the study, the results should not be interpreted as confirmatory; no adjustment for multiple tests was made. A *p*-value <0.05 was considered statistically significant. The analyses were performed using SAS, version 9.3 (SAS Institute, Chicago, IL).

## Results

**Fig. 1:**
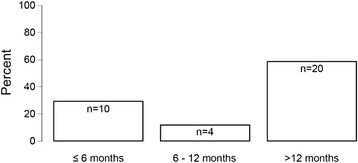
The frequency distribution of the time intervals between the first VTE episode and the diagnosis of the first tumor for the 34 patients in whom VTE preceded the diagnosis of cancer or both diagnoses coincided in time (*n* = 8). The median time interval (interquartile range) was 29.0 (0.0; 416.0) months

We identified 90 patients with PH (15.4 %) and 72 patients (12.3 %) with a history of at least 1 VTE at the time of referral, 48 of whom had a PE. Among the 45 patients with DVT, 93.3 % had leg DVT and 6.7 % had arm DVT. Only 10 patients reported recurrent VTE, and 3 patients developed VTE within 1 month prior to referral. All 3 patients had PE, and one of them had PH. In 51.3 % of the 72 patients, VTE was classified as cancer-related. The median time intervals (interquartile range) between the first VTE episode and referral were 43 (11; 134) months in the whole VTE group and 57 (25; 136) in the 19 patients with both VTE and PH. The time interval between the diagnosis of the first tumor and referral was 43 (7; 111) months in the whole cohort. Figure 1 shows the frequency distribution of the time intervals between the first VTE episode and the diagnosis of the first tumor for the 34 patients in whom VTE preceded the diagnosis of cancer or both diagnoses coincided in time.

**Fig. 2:**
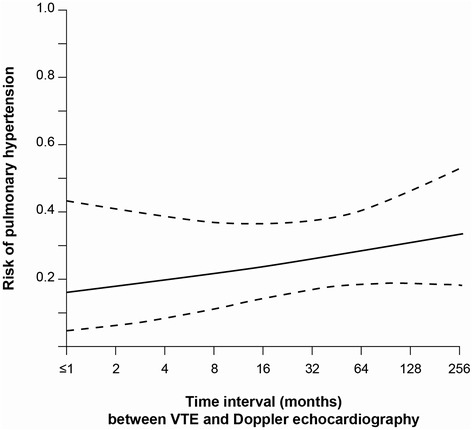
The unadjusted risk of pulmonary hypertension (solid line) with 95 % confidence intervals (dashed lines) among 72 cancer patients with venous thromboembolism (VTE), expressed as a function of the time interval between the first VTE episode and Doppler echocardiography. The time axis is plotted on a logarithmic scale to account for the wide range of time intervals and to better illustrate the effect of recent VTE on the risk of PH

Table 1 lists both the clinical data and the distribution of the pulmonary and cardiac findings in the subgroups, with and without VTE. The data for the 48 patients with documented PE is also given, but the between-group comparisons were limited to the VTE versus no VTE groups for the reasons indicated below. These two groups were well balanced, except for the prevalence of PH (26.4 *versus* 13.9 %, *p* <0.01). The odds of PH was higher in the subgroup with VTE than that without VTE in both the unadjusted analysis (OR 2.2, 95 % CI 1.2, 4.0) and the adjusted model (OR 2.4, 95 % CI 1.2, 4.5). Older age (OR 1.04, 95 % CI 1.0, 1.1) and the presence of severe airway obstruction (OR 2.6, 95 % CI 1.2, 5.4), atrial fibrillation (OR 2.1, 95 % CI 1.2, 3.8), significant mitral or aortic valve disease (OR 4.8, 95 % CI 2.4, 9.8) and left ventricular hypertrophy (OR 2.2, 95 % CI 1.2, 3.9) were also associated with an increased odds of PH, whereas the presence of impaired left ventricular function had no effect on the odds of PH (OR 0.7, 95 % CI 0.2, 1.9). Only 7 of the 35 patients with impaired left ventricular function had PH. When the predictor variable PE (*n* =48) was separately analyzed, the odds of PH increased by 3-fold in both the unadjusted (OR 3.1, 95 % CI 1.6-6.0) and the adjusted model (OR 3.3, 95 % CI 1.6, 6.8). The odds of PH did not depend on the time interval between the first VTE episode and referral (*p* = 0.32). The time course of the risk for PH is shown in Fig. 2.

At the time of referral, 38.1 % of the whole study group and 45.6 % of the 90 patients with PH had active malignant disease, which had no effect on the fully adjusted odds of PH (*p* = 0.12). The tumor-related prevalence values for both VTE and PE are included in Table 2. Advanced cancer was defined as active disease from solid tumors with at least N2 node involvement or distant metastases. Among the 72 patients with a history of VTE, 13 had advanced cancer [lung: 7, breast: 3, colon: 1, pancreas: 1, kidney: 1] at the time of referral. Advanced cancer was also diagnosed in 26 of the 90 patients with PH [lung: 17, breast: 2, colon: 2, pancreas: 2, ovary: 1, prostate: 1, mesothelioma: 1]. The prevalence values of PH according to 5 treatment groups are shown in Table 3.

**Table 2:** Prevalence of venous thromboembolism (VTE) and documented pulmonary embolism (PE) according to tumor group

	VTE (*n* = 68)	PE (*n* = 45)
Lung cancer	11/122 (9.0)	6/122 (4.9)
Breast cancer	16/113 (14.2)	8/113 (7.1)
Prostate cancer	6/57 (10.5)	5/57 (8.8)
Colorectal cancer	9/37 (24.3)	7/37 (18.9)
Other solid tumors	12/109 (11.0)	7/109 (6.4)
Lymphomas	8/67 (11.9)	7/67 (10.4)
Other hematologic malignancies	6/17 (35.3)	5/17 (29.4)

Figures in parenthesis are percentages. The analysis was limited to the 522 patients with one tumor entity

**Table 3:** Prevalence of pulmonary hypertension according to treatment group

Treatment group	Pulmonary hypertension
1: No previous antineoplastic chemotherapy or chest radiotherapy (*n* = 345)	53 (15.4 %)
2: Previous chemotherapy^a^ without anthracyclines (*n* = 114)	18 (15.8 %)
3: Previous chemotherapy with anthracyclines (*n* = 79)	11 (13.9 %)
4: Previous chest radiotherapy from 1995 onwards (*n* = 74)	10 (13.5 %)
5: Pre-1995 chest radiotherapy (*n* = 23)	8 (34.8 %)

^a^Alkylating agents, antimetabolites, microtubule-targeting agents, molecular-targeting agents. The treatment groups were not distinct from each other. Hence, no between-group comparisons could be made. The high proportion of patients with PH in group 5 probably relates to the high prevalence of atrial fibrillation and left heart disease among the 23 patients who had undergone pre-1995 radiotherapy (atrial fibrillation, 21.7 %; impaired left ventricular systolic function, 8.7 %; significant valvular heart disease, 34.8 %; left ventricular hypertrophy, 30.4 %)

## Discussion

Among cancer patients, a history of VTE was associated with a 2-fold increase in the odds of developing PH. Although it is tempting to emphasize the higher impact of PE on the risk of PH, we must acknowledge that the composite item VTE was selected as the primary predictor variable when designing this study for the following reasons: Treatment for VTE is usually initiated without requiring lung imaging when a DVT diagnosis is made via peripheral venous imaging. Therefore, several patients diagnosed with DVT only may also have developed PE. Conversely, a search for DVT is not mandatory in patients with documented PE because therapy of DVT and PE is the same. In the latter condition, the whole peripheral thrombus can dislodge from the venous wall and embolise. Both of these factors lead to an underestimation of the DVT prevalence among the VTE group.

The entry criteria of this study are critical in interpreting its results. The presence of pulmonary or cardiac symptoms was a prerequisite for entering the protocol. A patient with previous VTE who did not develop PH was more likely to be asymptomatic and was therefore less likely to be included in this study. Because of this referral bias, our study overestimated the strength of the relationship between a history of VTE and the presence of PH relative to the overall population of patients with both cancer and VTE which accounts for the high prevalence of PH among patients with VTE.

The effects of severe airway obstruction and older age on the odds of PH observed in this study were consistent with previous work. Pulmonary artery systolic pressure increases with age, and PH is common in the setting of advanced chronic obstructive pulmonary disease [10–12]. Pulmonary artery systolic pressure is also determined by the left ventricular filling pressure. Therefore, significant mitral or aortic valve disease, left ventricular hypertrophy and atrial fibrillation were each associated with a greater odds of PH. Atrial fibrillation may be a consequence of PH [13]. In this study group, however, there were only 8 patients with PH and atrial fibrillation who had neither left heart disease nor severe airway obstruction. Therefore, PH was the sole cause of atrial fibrillation in only a few of our patients. We unexpectedly observed no significant impact of impaired left ventricular systolic function on the odds of PH. Sample size problems may partially account for this negative result, which should be interpreted with caution. Only 35 patients (6.0 %) exhibited impaired left ventricular systolic function, although 98 patients (16.8 %) received anthracycline-based chemotherapy or pre-1995 radiotherapy of the chest, both of which are detrimental to the heart.

The association between a history of VTE and PH and a diagnosis of PH are less relevant in patients with a short life expectancy secondary to metastatic disease. In this study, only 18.1 % of the 72 patients with a history of VTE and 28.9 % of the 90 patients with PH had advanced cancer; therefore, our results are relevant to patients with less advanced active malignant disease or a history of cancer.

A definite PH diagnosis requires right heart catheterization, but this study could not have been completed using invasive techniques. This study relied on Doppler echocardiography to diagnose PH. A peak systolic pressure gradient across the tricuspid valve of 30 mmHg is considered the upper limit of normal [14]. To avoid diagnosing PH of questionable significance, we changed the threshold to 35 mmHg in this study. We did not add an estimated right atrial pressure to this gradient to approximate pulmonary artery systolic pressure because the estimation of right atrial pressure via the compressibility of the inferior vena cava is often unreliable among patients with advanced lung disease [15, 16]. In this study, the overall prevalence of lung disease was 62.1 %, and 9.7 % of patients had severe airway obstruction.

This monocenter study had further limitations. First, echocardiographic estimates of the peak systolic pressure gradient across the tricuspid valve may have been inaccurate due to poor visualization of the tricuspid regurgitation jet, particularly in patients with advanced chronic obstructive pulmonary disease. PH is not always accompanied by an analyzable tricuspid regurgitation jet; therefore, we missed several patients with this condition. Second, the imaging techniques for diagnosing and excluding of VTE are imperfect and vary in quality between institutions. However, as these two limitations apply to both patients with and without VTE, they tend to dilute the impact of VTE on the odds of PH and therefore paradoxically do not weaken the strength of our findings. Third, diastolic dysfunction contributes to PH and is common following mediastinal irradiation [17]. We did not include parameters pertaining to diastolic left ventricular function in the regression analysis because tissue Doppler imaging was not available at our institution before 2008. The assessment of transmitral flow curves via Doppler echocardiography cannot be used to reliably diagnose diastolic dysfunction. Given the significant effect of left ventricular hypertrophy on the odds for PH, we hypothesize that many of our patients with this condition had left ventricular diastolic dysfunction. Fourth, this study does not provide data on the prognostic impact of PH in cancer patients with previous VTE. Fifth, the small sample size of the PH subgroup precluded determining whether treatment-related factors interacted with the risk for PH, such as endocrine therapies, previous administration of chemotherapeutic agents implicated in lung injury, the time intervals between a wide range of chemotherapies and referral, and the type and duration of anticoagulant therapy following VTE. Similarly, the numbers of patients in the 6 tumor groups presented in Table 2 were too low for any attempts to determine the sites and stages of cancer that predispose to PH following VTE. Sixth, although all patients were interviewed for a history of VTE in a standardized manner and these data were checked with their medical records, we may have missed some patients who had been diagnosed with VTE at other institutions but did not recall that. Finally, we did not perform lung imaging tests on all patients with VTE and PH in this hypothesis-generating study; therefore, we lack data regarding the prevalence of findings suggestive of CTPH in this subgroup. This warrants further investigation.

Patients with unprovoked VTE are at an excess risk of receiving a subsequent diagnosis of cancer. Most tumors are diagnosed within the first 6 months after an episode of unprovoked VTE and the incidence rate of cancer declines to that in the general population at 12 months [18]. In our cohort, 58.8 % of the 34 VTEs preceding or coinciding with the diagnosis of the first tumor occurred more than 12 months before the cancer diagnosis was made, as shown in Fig. 1. However, it was impossible to ascertain from the historical data available to us how many of these VTEs were unprovoked, especially in patients with a history of remote VTE [19].

In the setting of an uncomplicated, acute PE, the risk of PH should rapidly decrease with time due to anticoagulant treatment. Figure 2 demonstrates that the risk of PH did not depend on the time interval between VTE and Doppler echocardiography. The association between a history of VTE and PH remained significant following the exclusion of the 3 patients with VTE within 1 month of referral. Therefore, transient PH secondary to uncomplicated PE cannot account for our findings. The median time interval of 43 months between VTE and referral is suggestive of the presence of a long-lasting obstruction within the pulmonary vascular bed following VTE. All patients included in this study had cardiac or pulmonary symptoms. It can be assumed that PH contributed to these symptoms in many patients with this condition. The long time interval between the diagnosis of the first tumor and referral observed in the 19 patients with VTE and PH [89 (46; 143) months] fits with registry data suggesting that many CTPH-associated cancers are survived several years before a diagnosis of CTPH is made [5].

In the general population, the cumulative incidence of CTPH is 3.8 % at 24 months following the first PE, with no further increase after more than 24 months of follow-up [20]. Given the limitations of echocardiography in the diagnosis of PH, this low incidence rate cannot explain the 3-fold increase in the odds of PH following PE observed in this study, even when the above-mentioned referral bias is taken into account. Beyond patient- and treatment-related features, a number of biological factors may account for a link between cancer and PH, such as the expression of hemostatic proteins, the production of microparticles by tumor cells, the presence of inflammatory cytokines, and the tumor cell expression of adhesion molecules that bind platelets which are activated in CTPH [21, 22]. Idiopathic pulmonary arterial hypertension was referred to as a localized form of cancer [23], because both conditions share several features at the cellular level including proliferation, hypertrophy and distal extension of smooth muscle cells, resistance to apoptosis, mitochondrial dysfunction, genomic instability and expression of cancer biomarkers [24, 25]. Cancer might predispose to an ongoing pulmonary vessel remodeling process that is initiated by VTE, maintained by the above-mentioned biological factors and results in persistent PH.

Our data suggest that PH after VTE is common among cancer patients with cardiac or pulmonary symptoms. Given the lack of a control group of non-cancer patients with cardiac or pulmonary symptoms and a history of VTE, however, this study provides no proof that this finding is specific for cancer patients. The odds of PH may be not be significantly lower in such a control group and the only difference may be the higher prevalence of VTE among the cancer patients reflecting the link between cancer and the activation of the hemostatic system.

Because of our findings we recommend to screen cancer patients with cardiac or pulmonary symptoms and a history of VTE for PH, even if the VTE episode occurred years before referral. Echocardiographic evidence of PH should raise concern regarding the presence of CTPH, particularly in younger symptomatic patients without the above-mentioned pulmonary or cardiac comorbidities associated with PH.

## Conclusion

The results of this study suggest a link between a remote or recent history of VTE and future PH in patients presenting with dyspnea, cough, chest pain, pulse irregularities, or exercise intolerance and either a history of cancer or active cancer. Our findings are not generalizable to non-cancer patients and cancer patients without pulmonary or cardiac symptoms after VTE due to referral bias towards an increased prevalence of PH in the VTE group of this study.

## Abbreviations

VTE: Venous thromboembolism
PH: Pulmonary hypertension
DVT: Deep venous thrombosis
CTPH: Chronic thromboembolic pulmonary hypertension
FEV1: Forced expiratory volume in 1 s
FVC: Forced vital capacity
OR: Odds ratio
CI: Confidence interval
